# Kv1.3 Channel Up-Regulation in Peripheral Blood T Lymphocytes of Patients With Multiple Sclerosis

**DOI:** 10.3389/fphar.2021.714841

**Published:** 2021-09-23

**Authors:** Ioannis Markakis, Ioannis Charitakis, Christine Beeton, Melpomeni Galani, Elpida Repousi, Stella Aggeloglou, Petros P. Sfikakis, Michael W. Pennington, K. George Chandy, Cornelia Poulopoulou

**Affiliations:** ^1^ National and Kapodistrian University of Athens, Medical School, Athens, Greece; ^2^ Department of Neurology, “St. Panteleimon” General State Hospital, Nikaia, Greece; ^3^ Department of Physiology and Biophysics, University of California, Irvine, Irvine, CA, United States; ^4^ Department of Molecular Physiology and Biophysics, Baylor College of Medicine, Houston, TX, United States; ^5^ Ambiopharm, Inc., North Augusta, SC, United States; ^6^ Lee Kong Chian School of Medicine, Nanyang Technological University, Nanyang Ave, Singapore

**Keywords:** T cells, potassium channels, Kv1.3, multiple sclerosis, patch clamp, calcium regulation

## Abstract

Voltage-gated Kv1.3 potassium channels are key regulators of T lymphocyte activation, proliferation and cytokine production, by providing the necessary membrane hyper-polarization for calcium influx following immune stimulation. It is noteworthy that an accumulating body of *in vivo* and *in vitro* evidence links these channels to multiple sclerosis pathophysiology. Here we studied the electrophysiological properties and the transcriptional and translational expression of T lymphocyte Kv1.3 channels in multiple sclerosis, by combining patch clamp recordings, reverse transcription polymerase chain reaction and flow cytometry on freshly isolated peripheral blood T lymphocytes from two patient cohorts with multiple sclerosis, as well as from healthy and disease controls. Our data demonstrate that T lymphocytes in MS, manifest a significant up-regulation of Kv1.3 mRNA, Kv1.3 membrane protein and Kv1.3 current density and therefore of functional membrane channel protein, compared to control groups (*p* < 0.001). Interestingly, patient sub-grouping shows that Kv1.3 channel density is significantly higher in secondary progressive, compared to relapsing-remitting multiple sclerosis (*p* < 0.001). Taking into account the tight connection between Kv1.3 channel activity and calcium-dependent processes, our data predict and could partly explain the reported alterations of T lymphocyte function in multiple sclerosis, while they highlight Kv1.3 channels as potential therapeutic targets and peripheral biomarkers for the disease.

## Introduction

Multiple sclerosis (MS) is a chronic autoimmune disorder of the central nervous system (CNS), characterized by white matter demyelination and intense perivascular infiltration by macrophages and auto-reactive T cells (Lassmann, 2004; Goverman, 2009; Gourraud et al., 2012) that migrate into the CNS and initiate myelin destruction, following their activation in the peripheral blood (PB) (Goverman, 2009; Severson and Hafler, 2010). With T cell activation being central in MS pathophysiology (Zhang et al., 1994), cellular components that control this process are of great importance and investigations concerning their expression and function could help the understanding of disease pathogenesis and the development of effective therapies.

Particularly relevant molecules in this context are the voltage-gated Kv1.3 channels, the main potassium conductance of T cells at rest and during the initial steps of their activation (Wulff et al., 2003; Cahalan and Chandy, 2009). Most importantly, these channels are part of the signalosome that clusters at the immunological synapse during antigenic stimulation (Panyi et al., 2003; Panyi et al., 2004), serving as key regulators of the calcium signaling required for cellular homeostasis and T cell activation. The activity of Kv1.3 channels, during the early events of T cell antigenic stimulation, determines the degree of membrane hyperpolarization and therefore the strength of the electrochemical driving force necessary for extracellular calcium entry into the cell via the calcium release activated calcium (CRAC) channels, the main calcium conductance of T cell plasma membrane (Cahalan and Chandy, 2009). Thus, Kv1.3 channel activity plays a central role in regulating the magnitude and duration of calcium responses during the initial steps of T cell activation and subsequently determines T cell fate and function (Lewis, 2001; Malissen and Bongrand, 2015).

Aside from their key role in T cell physiology, the interest in Kv1.3 channels in MS is further corroborated by a wealth of data associating these channels with the pathophysiology of the disease. Kv1.3 channels are up-regulated in activated effector memory T cells (T_EM_ cells) (Wulff et al., 2003; Cahalan and Chandy, 2009), a subset enriched in the myelin-specific T cell pool of MS patients. These cells specifically up-regulate Kv1.3 and not KCa3.1 channels, and are characterized as Kv1.3^high^KCa3.1^low^ (1,500 Kv1.3/cell and 50 KCa3.1/cell) (Wulff et al., 2003; Cahalan and Chandy, 2009). Interestingly, while quiescent myelin-specific T cells are present in the PB of both healthy individuals and MS patients (Zhang et al., 1994), activated Kv1.3^high^KCa3.1^low^ myelin-specific T_EM_ cells are only present in the PB of MS patients (Wulff et al., 2003). Additionally, inflammatory infiltrates in the lesions of MS brains are enriched in T_EM_ cells of the Kv1.3^high^ phenotype (Rus et al., 2005). The role of Kv1.3 channels in MS is further strengthened by reports showing that Kv1.3-selective blockers (Beeton et al., 2001; Beeton et al., 2006) and genetic knockout of Kv1.3 (Gocke et al., 2012), ameliorate disease in animal models of multiple sclerosis. Furthermore, a recent genetic study (Lioudyno et al., 2021) identified a link between a gain-of-function polymorphic allele of the Kv1.3 gene and rapid disease progression, more severe disease phenotype and increased PB CXCR3^+^ T_EM_ cells in MS patients.

Herein, we used a combination of patch clamp, flow cytometry and semi-quantitative PCR to study Kv1.3 channels in freshly isolated, unsorted, un-manipulated, non-cultured T cells from the PB of MS patients, patients with other neurological disorders (OND), patients with other inflammatory neurological disorders (OIND), patients with clinical isolated syndrome (CIS) and healthy controls with similar demographic characteristics. Our data demonstrate that resting T cells of patients with relapsing-remitting (RRMS) and secondary progressive MS (SPMS) manifest a significant increase in Kv1.3 expression, compared to healthy and disease controls. Moreover, it is shown, for the first time to our knowledge, that T cells from SPMS patients, express significantly higher numbers of functional Kv1.3 channels, compared to RRMS. Our findings suggest that Kv1.3 may serve as a useful peripheral biomarker for the disease, and support the use of Kv1.3 channel inhibitors for the treatment of MS and especially for SPMS, where treatment options are limited.

## Materials and Methods

### Patients and Controls- Greek Cohort

Study protocol had received prior approval by the Scientific and Ethics committee of St Panteleimon General State Hospital of Nikaia. Patients came from either the outpatient department or the neurological clinic of the above Hospital; Informed consent for blood sample collection as well as for clinical and demographic data use was obtained from all participating subjects.

Our study included 38 patients with definite MS according to the McDonald criteria (Thompson et al., 2018); twenty had relapsing-remitting and 18 secondary progressive disease. MS subtypes were defined according to clinical phenotypes (Lublin et al., 2014); a minimum of 12 months of gradual worsening was required to define SPMS. Nineteen (11 RRMS, 8 SPMS) patients were receiving immunomodulatory treatment during enrollment. Disability was measured using the Expanded Disability Status Scale (EDSS). All patients were enrolled in the Neurology department of St. Panteleimon General Hospital of Nikaia. Exclusion criteria comprised: evidence of MS relapse or corticosteroid treatment within the previous 30 days, active systemic bacterial, viral or fungal infection, coexisting systemic autoimmune disease, a history or presence of malignancy. Twenty-one healthy controls with similar demographic characteristics and similar exclusion criteria were also included. Additionally, 31 patients were enrolled as neurological controls: 16 patients with other neurological disorders (OND), 9 patients with other inflammatory neurological disorders (OIND) and 6 with clinical isolated syndrome (CIS) (Table 1).

**TABLE 1:** Characteristics of multiple sclerosis (MS) patients and controls. Greek cohort of MS patients and controls. *CIS*: Clinically Isolated Syndrome (3 optic neuropathy, 3 transverse myelitis). *OND*: Other Neurological Disorders (3 early-onset Alzheimer’s disease, 3 early-onset Parkinson’s disease, 2 motor neuron disease, 3 ischemic stroke, four epileptic seizures, one Vitamin B12 deficiency). *OIND*: Other Inflammatory Neurological Disorders (2 limbic encephalopathy, five acute lymphocytic meningitis, one lupus-associated myelopathy, one neuro-Behçet syndrome). Values are expressed as means ± SD.

Disease	*n*	Sex (M/F)	Age (years)	Treated/untreated	Disease duration (years)	EDSS
**MS**	38	16/22	37.2 ± 7.4	26/12	8.5 ± 5.7	3.5 ± 1.8
*Relapsing remitting*	20	10/11	36.2 ± 7.2	13/7	6.7 ± 4.8	2 ± 1.2
*Secondary progressive*	18	7/11	38.4 ± 7.6	13/5	10.9 ± 5.8	4.83 ± 1.3
**CIS**	6	2/4	38.5 ± 3.8	2/4	2.8 ± 1.5	1.5 ± 1.4
**OND**	16	9/7	40.9 ± 10.8	—	—	—
**OIND**	9	5/4	39.6 ± 4.2	9/0	1,3 ± 0,8	—
**Healthy controls**	21	10/11	38.1 ± 8	—	—	—

### Patients and Controls–US Cohort

In a previous study, we analyzed peripheral blood T cells from unrelated, non-Hispanic Caucasians with clinically definite MS. Patients were obtained from the MS clinic at the University of California Irvine; age- and sex-matched healthy controls were also included in the study (Mkhikian et al., 2011). Here, archived residual peripheral blood T cells from that study (9 MS patients and 22 healthy individuals) (Table 2) were used for flow cytometry to characterize cell surface K_V_1.3 expression.

**TABLE 2:** Characteristics of multiple sclerosis (MS) patients and controls. North American Cohort of MS patients. *ND*: Non-disclosed, *FH:* Family History; *AID:* Autoimmune Disease; *AITD*: autoimmune thyroid disease.

Patient ID	Age	Gender	MS type	FH of AID	Rx
Pa1	35	F	RR MS	No	No
Pa2	51	M	RR MS	No	No
Pa3	46	F	RR MS	MS	No
Pa4	ND	F	RR MS	ND	No
Pa5	75	F	SP MS	MS	No
Pa6	40	M	PP MS	No	No
Pa8	48	F	RR MS	No	No
Pa11	47	F	MS	ND	?
PaJ	67	F	PP MS	MS, AITD	No

### Cell Preparation

Mononuclear cells were isolated from heparinized whole blood by Ficoll-Hypaque density gradient centrifugation (Boyum, 1968). The isolated buffy coat was washed 3X with PBS without Ca^2+^ and re-suspended in RPMI 1640 culture medium and monocytes (CD14^+^ cells) and B cells (CD19^+^) were depleted by the use of magnetic beads (Dynabeads CD14 and CD19 ThermoFisher). The isolated T cells were separated into two pools: one for electrophysiology experiments on the same day of isolation and the second for RNA isolation.

### Solutions

The standard extracellular solution used in patch clamp experiments contained 135 mM NaCl, 5mM KCl, 1 mM CaCl_2_,1 mM MgCl_2_, and 10 mM HEPES, pH 7.4; osmolality, 280 mOsmol/kg). The standard pipette solution was composed of 100 mM KCl, 40 mM KF, 1 mM CaCl_2_, 1 mM MgCl_2_, 10 mM EGTA, and 10 mM HEPES, pH 7.4; osmolality, 290 mOsmol/kg), having low calcium concentrations, well below the K_Ca_ channel activation threshold (Poulopoulou et al., 2008). Pipettes were filled with solutions filtered through a 0.22 µm-syringe filter (Thermo Scientific). Modified solutions were also used in some experiments and their composition is stated in the text.

### Electrophysiological Recordings

All patch clamp experiments were performed in the whole-cell configuration. We focused on patch clamping small size (resting) T cells (average cell capacitance 1.6 pF) and avoided the enlarged ones. Membrane currents were measured in the whole-cell configuration of the patch clamp technique, as previously described (Poulopoulou et al., 2005a). Membrane currents were corrected for liquid junction potentials. Pipettes were fabricated from R-series Borosilicate Glass Capillaries (World Precision Instruments, Sarasota, FL) using a two-stage puller (L/M-3P-A; List Medical) and had resistances between 3 and 5 MΩ. All experiments were performed at room temperature (20–25°C). Currents were low-pass filtered at 2 kHz. Kv1.3 currents were recorded in response to 150 ms voltage ramps from −100 to +100 mV, out of a holding potential of −90 mV. Peak currents were also measured from the same holding potential, in response to 1000-ms voltage-steps from −80 to +40 mV, in 15 mV increments, given every 60 s, and conductance-to-voltage curves (g-V) were constructed using the chord equation (Hille, 2001). The voltage dependence of steady-state inactivation was estimated by clamping the cell membrane at -90 mV and then stepping to various pre-pulse potentials (−120 to 0 mV, in 15-mV increments) for 100 s and then to a test pulse of +40 mV for 100 ms. Current measurements were performed using the pClamp software (Axon Instruments). The majority of T cells studied were resting T cells (cell capacitance 1.6 pF). To normalize for cell size, we determined channel density/μm^2^ of cell surface area, by dividing the average channel number/cell by the average cell surface area, determined from cell capacitance measurements (1 pF = 100 μm^2^). Data were further analyzed using Origin technical graphics and data analysis program (OriginLab Corp., Northampton, MA). Peak conductance-to-voltage and steady-state inactivation curves were fitted to Boltzmann functions. Kinetic parameters were calculated by fitting whole-cell currents to the Hodgkin-Huxley n4 j kinetic model (Cahalan, et al., 1985).

### RNA Preparation, Reverse Transcription, and Polymerase Chain Reaction

Total RNA isolation and cDNA synthesis were performed as follows (Poulopoulou et al., 2005b): 1 µl of cDNA synthesized by 2 μg of total RNA, extracted using the RNAeasy kit (Qiagen, Chatsworth, CA) following the manufacturer’s instructions. The PCR amplification was performed in a final volume of 50 µl (1xPCR Buffer, 200 µl of each dNTP, 25 pmol of each primer and 2.5 µl of HotStarTaq DNA Polymerase (Qiagen). All samples used had produced negative results when total RNA was used instead of cDNA, confirming the absence of DNA contamination from the sample. The efficacy and the integrity of the cDNA in all our samples were assessed by the amplification of the housekeeping gene glyceraldehyde-3-phosphate dehydrogenase (GAPDH). The initial denaturation step of the PCR was performed at 94°C for 15 min followed by 35 cycles at 94°C for 1 min for Kv1.3, TASK-2 and KCa3.1 gene and 30 cycles for GAPDH gene, at the appropriate annealing temperature (54^ο^ C for all four genes) for 1 min and at 72°C for 1 min. The last cycle was followed by a final extension step at 72°C for 10 min. The PCR products were subjected to electrophoresis in 1.5 (w/v) agarose gels.

PCR products that were run in 1.5 (w/v) agarose gels demonstrated: one band with a molecular size of 103 bp, corresponding to K_V_1.3 transcripts, one band with a molecular size of 106 bp, corresponding to TASK-2 transcripts, one band with a molecular size of 125 bp, corresponding to KCa3.1 transcripts and one band with a molecular size of 117 bp, corresponding to GAPDH transcripts.

The intensity of each band was measured by densitometric scanning, and the value of each Kv1.3, TASK-2 and KCa3.1 band from each sample was normalized to the intensity value of the housekeeping gene Glyceraldehyde-3-Phosphate Dehydrogenase (GAPDH) (Poulopoulou et al., 2005b). The RefSeq accession number, the sequence of the PCR primers, the number of cycles and the amplified fragment size, were as follows:

Kv1.3: (NM_002232.5) F:5’-GTGTCTTGACCATCGCATTG-3’, R:5’-ACGTGCATGTACTGGGATTG-3’, 35 cycles, bp:103; TASK-2: (NM_003740.4) F:5’-TGGTGATCCCACCCTTCGTA -3’, R: 5’-ACA​AAG​TCA​CCG​AAG​CCG​AT-3’, 35 cycles, bp:106; KCa3.1 (NM_002250.3): F:5’-ATGCAGAGATGCTGTGGTTCG-3’,R:5’-GACCTCTTTGGCATGAAAGGC-3’, 35 cycles, bp:125; GAPDH: (NM_002046.7) F:5-CTCCAAAATCAAGTGGGGCG-3, R:5’-ATGATGACCCTTTTGGCTCCC -3’, 30 cycles, bp:117.

Human brain total RNA was purchased from TAKARA BIO and was used as our positive internal control for all primers.

### Flow Cytometry Experiments

K_V_1.3 expression was detected on peripheral blood mononuclear cells by flow cytometry using a validated fluorescein-conjugated ShK-F6CA assay. ShK-F6CA is a highly specific K_V_1.3 inhibitor that blocks the channel with low picomolar affinity and binds to the channel tetramer (Beeton et al., 2003). Peripheral blood T cells were washed twice with phosphate buffered saline (PBS) and incubated in the dark at room temperature with 100 nm ShK-F6CA in PBS +2% goat serum (Sigma) for 30 min and then washed 3× with PBS +2% goat serum before flow cytometry analysis. Stained mononuclear cells were analyzed by flow cytometry on a BD Biosciences FACScan as described previously (Beeton et al., 2003). Data were further analyzed using CellQuest software. For each subject, staining intensity was compared between cells stained with ShK-F6CA versus unlabeled ShK using the Kolmogorov-Smirnov Test (Cell Quest software) to subtract autofluorescence (D values).

### Statistics

Statistical analysis was performed using Student’s t test with an accepted level of *p* < 0.05. For multiple comparisons one-way analysis of variance was applied with Bonferroni *post hoc* correction. Normality of data was assessed using the Kolmogorov-Smirnov test. The goodness of data fit to exponential or Boltzmann functions was evaluated with the Hamilton’s R coefficient. All results presented in the text are mean ± standard error.

## Results

### T Lymphocytes of MS Patients Exhibit Higher Kv1.3 Currents Compared to Healthy and Disease Controls

Previous studies of MS patients measured Kv1.3 expression in antigen-specific T cell lines or MHC-tetramer-sorted T cells that had been activated and expanded through repeated *in vitro* antigenic stimulations before patch clamp analysis (Wulff et al., 2003; Beeton et al., 2006). Here, we performed whole-cell patch clamp experiments in negatively selected, freshly isolated T cells from MS patients and various control groups in order to investigate the biophysical characteristics of Kv1.3 currents and calculate the number of functional channels/surface area of each cell.

**FIGURE 1:**
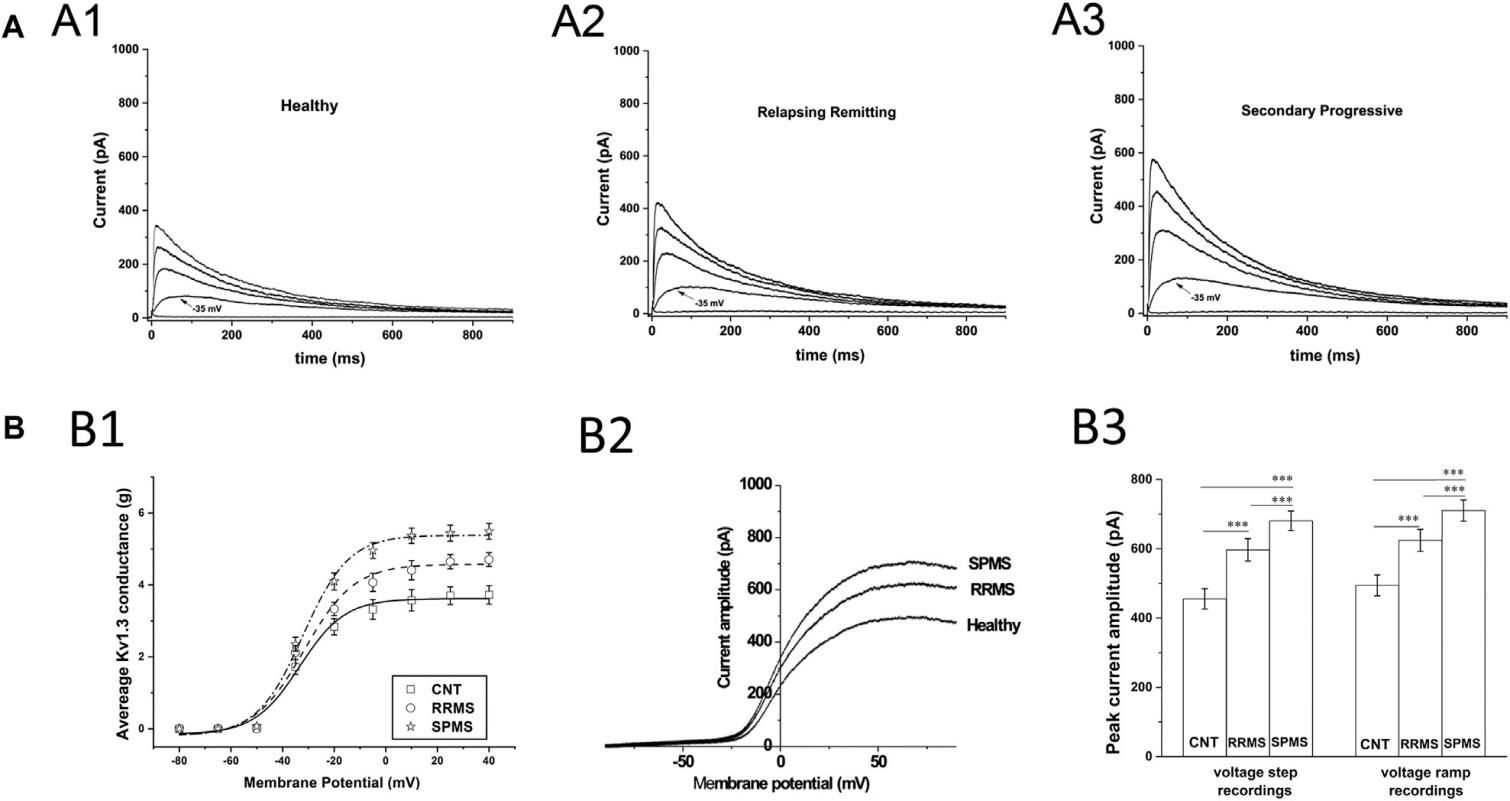
Patch clamp data from T cells of MS patients and healthy controls. **(A)**: Higher current responses in MS T cells. Whole-cell currents from T lymphocytes bathed in extracellular solution of pH 7.4 with their membrane clamped at −90 mV. MS T cells (N = 855) have higher peak K_V_1.3 amplitudes than controls (N = 1,258). **(** **A1–A3):** Representative K_V_1.3 currents elicited by 1000-ms depolarizing pulses in 15-mV increments, given every 60 s are displayed at physiologically relevant potentials (−50 to +10 mV) for reasons of clarity. **(B)**: T cell Kv1.3 currents in SPMS have significantly higher mean peak amplitudes than both RRMS and healthy controls. **(B1)**: Increased peak Kv1.3 conductance, in MS patients compared to healthy individuals. Peak current amplitudes in response to voltage step protocols were converted to conductance (g) values, averaged and plotted against the corresponding voltage for SPMS (* ), RRMS (○ ) and healthy controls (□ ) (g-V curves). The average maximal peak conductance was 5.49 ± 0.22 for SPMS (*n* = 18 patients, N = 415 cells), 4.7 ± 0.19 for RRMS (*n* = 20 patients, N = 440 cells) and 372 ± 0.25 for healthy individuals (*n* = 21, N = 542 cells) (*p* < 0.001). Data shown are average values ±S.E. Plots are fitted to Boltzmann functions. **(** **B2):** Representative whole-cell currents of a healthy individual, an RRMS patient and a SPMS one, in response to voltage-ramps (−100 to +100 mV). Both RRMS and SPMS T cells have higher peak amplitudes than T cells of their control counterparts, while SPMS T cells present higher peak amplitudes than the RRMS patients **(B3)**: **Left**: Average peak Kv1.3 currents, in response to +40 mV voltage steps, were 680.5 ± 28.4 for SPMS (*n* = 18 patients, N = 415 cells), 596.7 ± 32.2 for RRMS (*n* = 20 patients, N = 440 cells) and 455.3 ± 29.5 for healthy controls (*n* = 21, N = 542 cells) (٭٭٭: *p* < 0.001). **Right**: Average peak currents in response to voltage ramps were 710.21 ± 29.9 for SPMS (*n* = 18 patients, N = 1,410 cells), 624.1 ± 31.5 for RRMS (*n* = 20 patients, N = 1,480 cells) and 494.3 ± 30.2 pA for healthy controls (*n* = 21, N = 1,510 cells) (٭٭٭: *p* < 0.001). One way ANOVA with Bonferroni post hoc correction was applied for statistical analysis.

We recorded outward potassium currents in response to voltage ramp protocols (*see methods section*) (N = ∼70 T cells/subject). In a subset of these cells (N = ∼20 T cells/subject) we also recorded currents in response to: 1) voltage-step activation and 2) steady-state inactivation protocols (*see methods section*). Cell capacitance was constantly monitored. Currents were identified as Kv1.3 based on the presence of cumulative inactivation, a distinctive property of Kv1.3 and their total inhibition by 1 nM of the Kv1.3-selective blocker ShK-186 (Matheu et al., 2008). Kinetic measurements of Kv1.3 current activation and inactivation and calculated whole-cell activation and steady-state inactivation conductance-to-voltage relations showed that Kv1.3 currents from MS T lymphocytes have similar biophysical characteristics with currents recorded from either healthy individuals or patients with other neurological disorders (Table 3). Interestingly, T lymphocytes isolated from MS patients (*n* = 38) exhibited currents with significantly higher peak amplitudes than those of healthy (*n* = 21) and disease controls (*n* = 31) (Figures 1A,B). Given that Kv1.3 currents from both MS patients and controls have similar gating and kinetic properties, it follows that the increased current amplitude in MS T lymphocytes is due to up-regulation in the number of Kv1.3 channels.

**TABLE 3:** Electrophysiological properties of Kv1.3 channels, in patients with multiple sclerosis (MS) (*n* = 38) and control groups (*n* = 52). Data are presented as means ± SEM. (t_n_: activation time constant; t_j_: inactivation time constant; V_n_: potential of half-maximal activation; V_j_: potential of half-maximal steady-state inactivation; I_max_: peak current amplitude following depolarization to +40 mV; C_m_: membrane capacitance).

Group	Activation threshold	τ_n_ (ms)	τ_j_ (ms)	V_n_ (mV)	V_j_ (mV)	I_max_ (pA)	C_m_ (pF)	Kv1.3 channel density (channels/μm^2^)
Definite MS	−35 mV	3.4 ± 0.4	169 ± 4.2	−32.3 ± 2.5	−53.5 ± 1.9	636.3 ± 29.3	1.67 ± 0.1	2.5 ± 0.04
Relapsing remitting	−35 mV	3.5 ± 0.3	167 ± 3.2	−31.8 ± 2.1	−53.1 ± 2.2	596.7 ± 32.2	1.68 ± 0.1	2.4 ± 0.04
Secondary progressive	−35 mV	3.4 ± 0.5	171 ± 5	−32.7 ± 2.6	−53.7 ± 2.1	680.5 ± 28.4	1.65 ± 0.2	2.7 ± 0.05
CIS	−35 mV	3.5 ± 0.3	169 ± 4.7	−31.9 ± 2.5	−52.8 ± 2.3	432.6 ± 36.8	1.80 ± 0.1	1.8 ± 0.09
OND	−35 mV	3.6 ± 0.5	172 ± 5.1	−32.3 ± 3	−51.6 ± 1.9	449.2 ± 35.1	1.6 ± 0.2	1.8 ± 0.0.7
OIND	−35 mV	3.8 ± 0.4	173 ± 4.4	−32 ± 2.4	−51.9 ± 2	435.66 ± 31.9	1.68 ± 0.1	1.8 ± 0.1
Healthy controls	−35 mV	3.5 ± 0.3	171 ± 4.4	−33.1 ± 2.6	−53.5 ± 1.8	455.3 ± 29.5	1.65 ± 0.1	1.8 ± 0.05

### Channel-Specific Protocols Exclude TASK-2 Contribution in the Potassium Current Up-Regulation of MS T Lymphocytes

**FIGURE 2:**
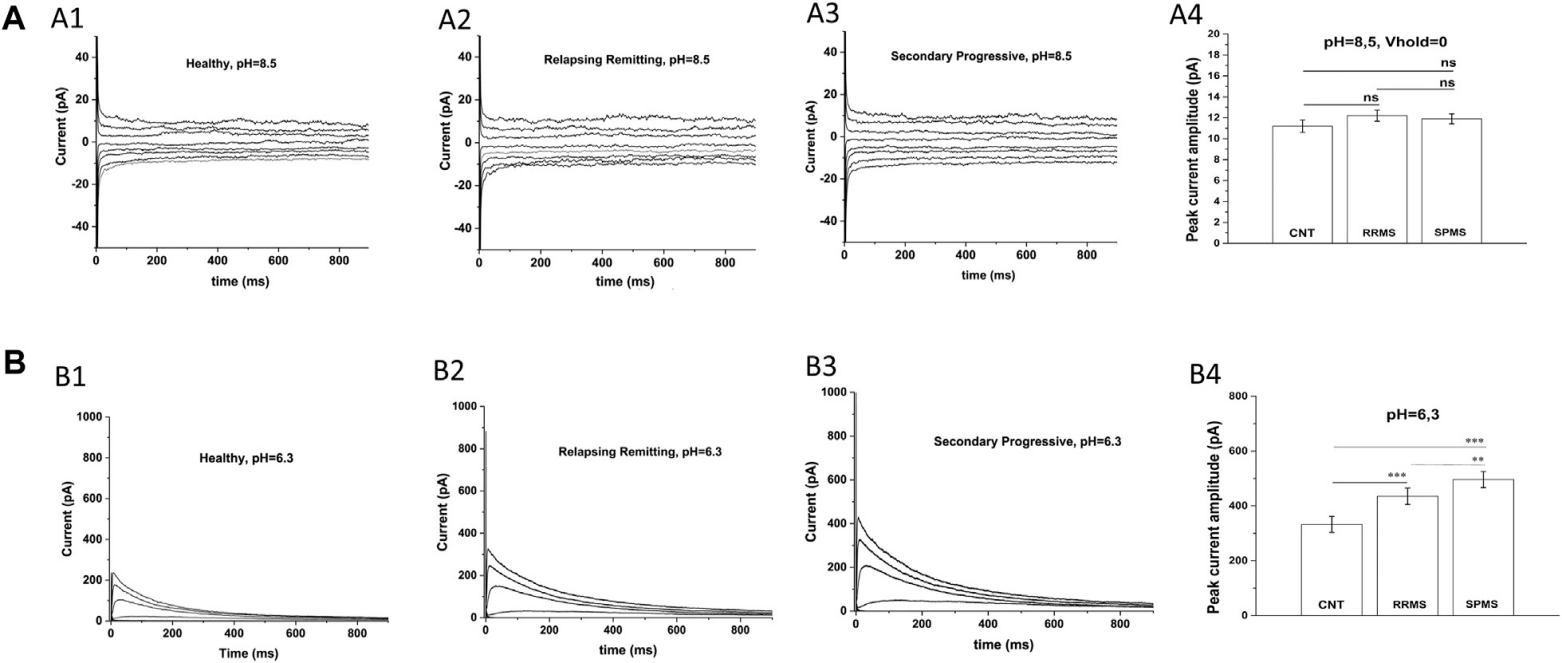
Specific electrophysiological protocols exclude TASK-2 contribution to the increased potassium conductance of MS T lymphocytes. **(A)**: T cells from either MS patients or healthy controls present similar current-responses in alkaline solution at a 0 mV holding potential: Whole-cell currents recorded from T lymphocytes bathed in an alkaline (pH = 8.5) extracellular solution with their membranes clamped at 0 mV. **(** **A1–A3):** Whole-cell current responses to 15 mV voltage-steps, given every 5 s from T lymphocytes of healthy controls, RRMS and SPMS patients. **(** **A4):** Average peak currents (at +40 mV) did not differ significantly between groups. Average values ±SE were: 11.9 ± 0,48 pA for SPMS (*n* = 12, N = 235 cells), 12.2 ± 0,53 pA for RRMS (*n* = 14, N = 282 cells) and 11.2 pA ± 0,6 for healthy subjects (*n* = 15, N = 310 cells) (ns: non-significant). Method: One way ANOVA with Bonferroni post hoc correction. **(B)**. T cells of MS patients exhibit higher K^+^ currents than controls in acidic extracellular solution: Whole-cell currents recorded from T cells bathed in acidic (pH = 6.3) extracellular medium with their membrane held at −90 mV. K_V_1.3 currents from T cells of healthy controls, RRMS and SPMS patients elicited by 1000-ms depolarizing pulses in 15-mV increments, given every 60 s (pH = 6.3). **(B1–B3):** Displayed are whole-cell K_V_1.3 current responses at physiologically relevant potentials, ranging from –50 mV to +10 mV. T cells from MS patients bathed in acidic extracellular conditions present higher current responses than controls. **(B4)**: Peak currents at +40 mV were: 496.06 ± 34.5 for SPMS (*n* = 12, N = 230 cells), 435.08 ± 32.1 pA for RRMS (*n* = 12, N = 252 cells) and 332.36 ± 28.9 pA for healthy controls (*n* = 14, N = 310 cells) (٭٭٭: *p* < 0.001, ٭٭: *p* < 0.01, one-way ANOVA).

An earlier study (Bittner et al., 2010) has also reported increased potassium currents in T cells of MS patients but they attributed this up-regulation to pH-sensitive TASK-2 channels, rather than Kv1.3 channels. For this reason, we examined the potential contribution of TASK-2 conductance to our potassium current recordings by taking advantage of the very distinct biophysical properties of these channels. TASK-2 channels are voltage-independent, non-inactivating, do not exhibit cumulative inactivation, and their opening is facilitated by alkaline pH (Morton et al., 2005). Thus, we exposed T cells to an alkaline extracellular solution (pH 8.5) to enhance opening of TASK-2 channels, and used a 0 mV holding potential to inactivate all Kv1.3 channels–(Hajdu et al., 2010) without impacting TASK-2 channels (Morton et al., 2005). As seen in Figure 2A currents elicited under these conditions had very small amplitude and did not differ between MS patients (11.9 ± 0.48 pA for SPMS *n* = 12, 12.2 ± 0.53 pA for RRMS *n* = 14) and healthy controls (11.2 ± 0.6 pA *n* = 15). These small currents were voltage-independent, non-inactivating, and had a reversal potential of 5 mV which is close to the reversal potential of chloride (0 mV) or of non-selective cations (0 mV) rather than potassium (−83.5 mV) (Figure 2A); we did not further characterize these currents because it was out of the scope of this investigation. Therefore, under TASK-2 favoring conditions, we were unable to detect any measurable TASK-2 conductance in T cells of either MS patients or healthy individuals. This is in line with previous studies that also did not detect TASK-2 currents in T cells (Hajdu et al., 2010), and reported Kv1.3 and calcium-activated channels KCa3.1 as the only functional potassium channels in human and rat T cells (Chiang et al., 2017).

Next, we recorded currents from T lymphocytes bathed in acidic extracellular solution (pH 6.3), where TASK-2 activation is minimal (Morton et al., 2005) and if present their contribution will be negligible. Currents were elicited from a holding potential of −90 mV in response to membrane depolarizations delivered every 60 s. Under these TASK-2 hindering conditions, T cells exhibited currents with biophysical properties characteristic of Kv1.3 (fast activation, slow inactivation and cumulative inactivation), and with amplitudes significantly higher in MS (496.06 ± 34.5 pA for SPMS *n* = 12, 435.0811.9 ± 32.1 pA for RRMS *n* = 12) than control T cells (332.36 ± 28.9 pA *n* = 14) (Figure 2B *p* < 0.001). These results corroborate and support our data obtained with regular extracellular solutions (pH 7.4) and validate the characterization of our currents as *bona fide* Kv1.3. Taken together, our findings indicate that PB T lymphocytes from MS patients express Kv1.3 currents with significantly higher amplitude than PB T cells from controls (680.5 ± 28.4 for SPMS vs 596.7 ± 32.2 for RRMS and 455.3 ± 29.5 for CNTs at pH 7.4).

### Kv1.3 Channel Membrane Density is Increased in MS T Lymphocytes

**FIGURE 3:**
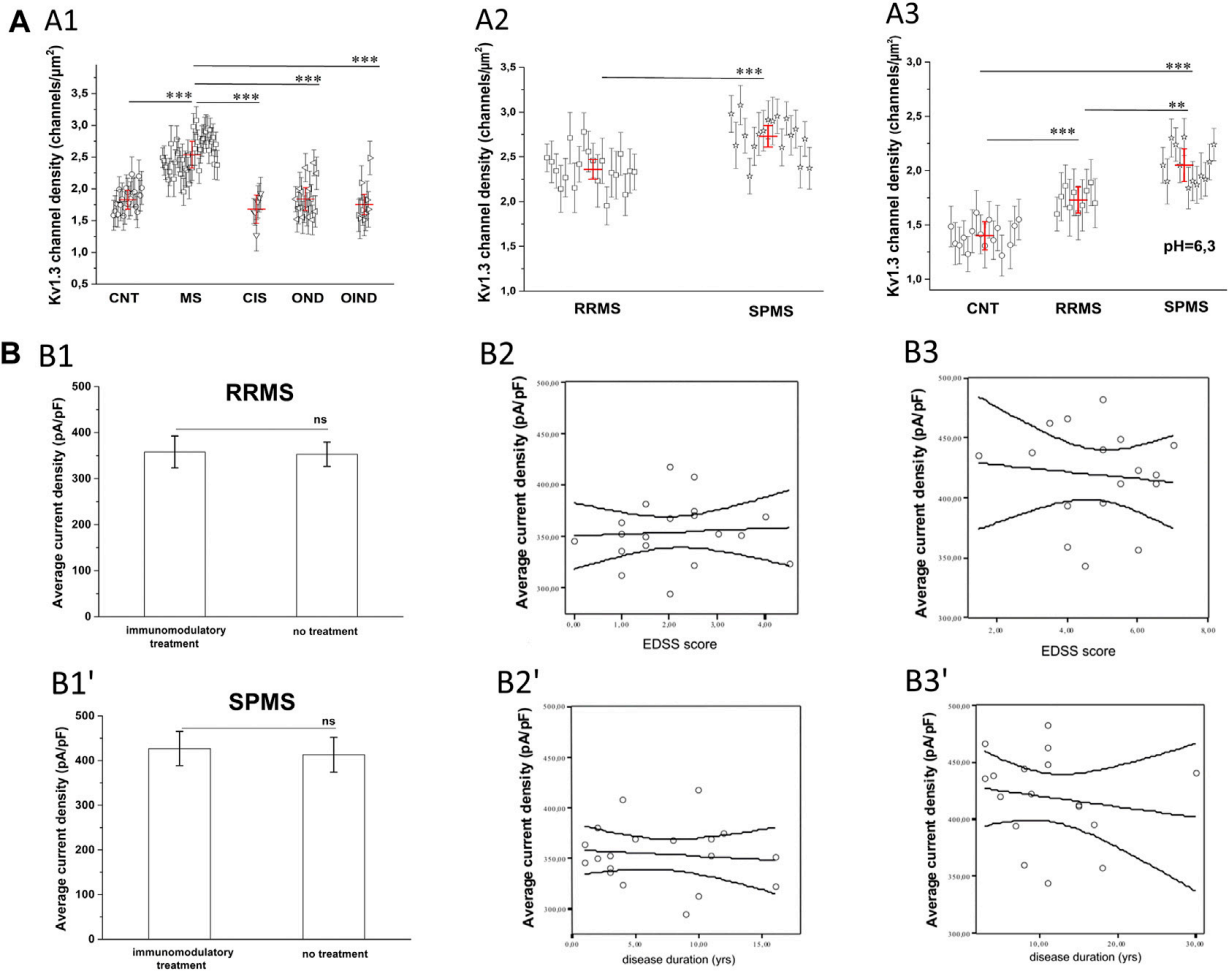
**(A)**: Calculated K_V_1.3 channel densities per T lymphocyte from whole-cell currents recorded from a holding potential of −90 mV. **(A1):** K_V_1.3 channel densities in MS patients (*n* = 38, N = 852 cells) are significantly higher (*p* < 0.001), compared to healthy control subjects (*n* = 21, N = 540 cells), patients with: clinically isolated Syndrome (CIS) (*n* = 6, N = 130 cells), other neurological disorders (OND) (*n* = 16 patients, N = 335 cells), and other inflammatory neurological disorders (OIND) (*n* = 9 patients, N = 253 cells). Each symbol represents mean values for each individual patient and error bars are standard deviation of the mean. Red horizontal lines with red error bars correspond to the average value ±standard error for each group. Comparisons are shown only for statistically significant differences (٭٭٭: *p* < 0.001). One way ANOVA with Bonferroni post hoc correction was applied for statistical analysis. **(** **A2):** Sub-grouping of MS patients showed significantly higher K_V_1.3 channel densities in Secondary Progressive Multiple Sclerosis (SPMS) (*n* = 18 patients, N = 415 cells), compared to Relapse Remitting Multiple Sclerosis (RRMS) (*n* = 20 patients. N = 440 cells). Values shown are mean ± SD for each subject. Red horizontal lines correspond to the average value ±standard error for each group. (*p* < 0.001). (٭٭٭: *p* < 0.001). **(A3)** In acidic extracellular solution, where TASK-2 activation is negligible, Kv1.3 channel densities are significantly higher in T cells of patients with SPMS (*n* = 12, N = 230 cells), compared to both RRMS (*n* = 12, N = 252 cells) and healthy controls (*n* = 14, N = 310 cells). Individual marks represent mean ± SD for each subject; red horizontal lines and bars represent average ±SE. (٭٭٭: *p* < 0.001, ٭٭: *p* < 0,01). **(B)**. Effect of MS severity (MMSE score), duration (years) and immunomodulatory treatment on K_V_1.3 current density. **(B1-B1′)**. K_V_1.3 density was not affected by immunomodulatory treatment in RRMS [treated: 357.7 ± 34 pA/pF, (*n* = 14); untreated: 350.2 ± 27 pA/pF, (*n* = 6),] or SPMS patients [treated: 426.8 ± 38.3 pA/pF (*n* = 13); untreated: 412.9 ± 39 pA/pF, (*n* = 5)]. Average values ±SE are shown (ns: non-significant). **(** **B2–Β2′).** In RRMS, K_V_1.3 current density did not correlate with either EDSS (r^2^ = 0.05, *p* = 0.7) or disease duration (r^2^ = 0.1, *p* = 0.3). **(** **B3-Β3′).** In SPMS, correlations were also non-significant for either EDSS (r^2^ = 0.11, *p* = 0.5) or disease duration (r^2^ = 0.16, *p* = 0.6).

The finding that PB T lymphocytes of MS patients manifest higher Kv1.3 currents could be due to either a larger cell size or an increase in the membrane density of Kv1.3 channels. Based on cell capacitance measurements, T lymphocyte sizes were similar in MS patients (average Cm = 1.67 pF) and healthy controls (average Cm = 1.65 pF). The above capacitance values indicate that recordings were made from resting rather than activated T cells. Furthermore, calculation of the Kv1.3 channel densities/μm^2^ of cell surface, as described in the *methods* section, showed that T cells had significantly higher Kv1.3 channel densities in MS, (*n* = 38, N = 852 cells) compared to healthy individuals (*n* = 21, N = 540 cells), or patients with OND (*n* = 16, N = 335 cells), CIS (*n* = 6, N = 130 cells) and OIND (*n* = 9, 253 cells) (Figure 3A1, *p* < 0.001). The higher Kv1.3 channel density in MS is not because a minority of cells (e.g., activated TEM cells with a Kv1.3^high^ phenotype) expressed high numbers of Kv1.3 channels and skewed the average. Of 855 MS-patient T cells studied with voltage step protocols, 86% had a channel density greater than 2.1 channels/µm^2^, whereas only 15% of 1,258 control T cells studied (healthy individuals, OND, OIND, CIS) had a channel density above this cut-off value. Therefore, these results indicate that the majority of resting T-cells in MS exhibit an elevated Kv1.3 channel density and suggest that Kv1.3 expression may be a useful biomarker to distinguish MS from other CNS inflammatory disorders.

Sub-grouping of MS patients into relapsing-remitting (RRMS) and secondary progressive (SPMS) disease revealed that T lymphocytes of SPMS patients had significantly higher Kv1.3 current amplitudes (Figure 1B, *p* < 0.001) and channel membrane density (Figure 3A2, *p* < 0.001) than T lymphocytes of RRMS patients. Similar results were obtained from experiments performed in acidic (6.3) pH, where, as aforementioned, TASK-2 activation is minimal. Under these conditions T lymphocytes from SPMS patients had significantly higher Kv1.3 densities compared to both RRMS patients and healthy controls (Figure 3A3).

For both RRMS and SPMS subgroups, there was no significant correlation between Kv1.3 channel density and either immunomodulatory treatment (Figures 3B1,B1′), disability or disease duration (Figures 3B2,B2′,tB3,B3′). Therefore, increased Kv1.3 expression in T cells is correlated to the subtype of MS rather than any other parameter we tested.

### Enhanced Kv1.3 mRNA Expression in T Lymphocytes From MS Patients Compared to Controls

**FIGURE 4:**
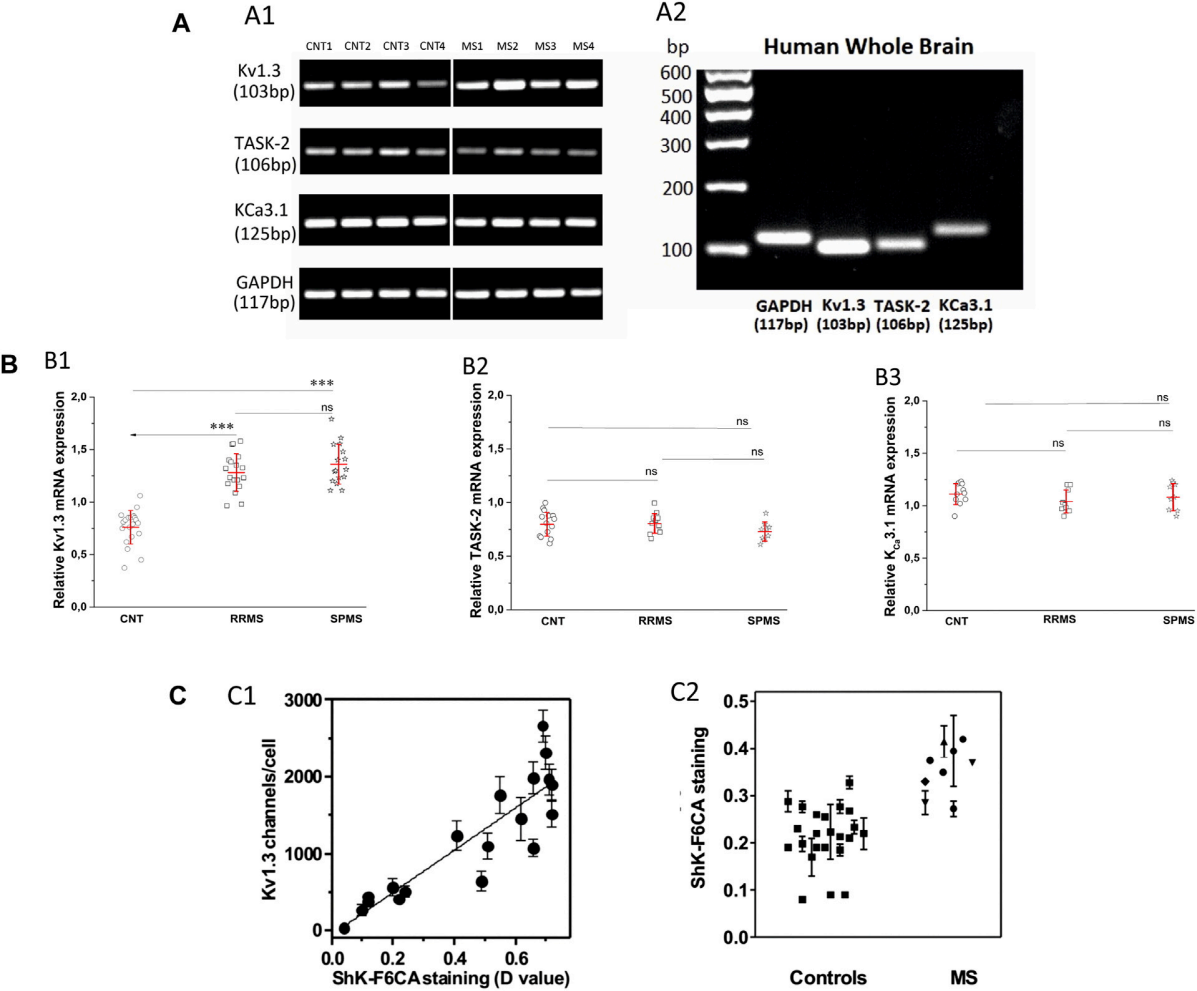
**(A)**. Expression of Kv1.3, TASK-2 and KCa3.1 mRNA in T lymphocytes of patients with multiple sclerosis. **(A1):** Agarose gel image from PCR products of K_V_1.3, TASK-2, KCa3.1 and the housekeeping gene glyceraldehyde-3-phosphate dehydrogenase (GAPDH) in T lymphocytes of four healthy control subjects (lanes CNT 1–4) and four patients with multiple sclerosis (lanes MS 1–4). The PCR products demonstrated: a band with a molecular size of 103 bp, corresponding to K_V_1.3, a band of 106 bp corresponding to TASK-2, a band of 125 bp corresponding to KCa3.1, and a band of 117 bp corresponding to GAPDH transcripts. MS patients gave K_V_1.3 bands of higher intensity (1.32 ± 0.18, *n* = 34) compared to healthy controls (0.76 ± 0.16, *n* = 20, *p* < 0.001) and similar intensities for the TASK-2 (controls 0.78 ± 0.12, MS 0.77 ± 0.1) and KCa3.1 (control 1.12 ± 0.14, MS 1.06 ± 0.11) bands in these samples. In the same samples the bands of the housekeeping gene GAPDH had similar intensities.** (** **A2):** Agarose gel image of the PCR products of GAPDH, Kv1.3, TASK-2 and KCa3.1 amplified from cDNA derived from commercially available human whole brain (HWB) RNA. HWB was used as positive internal control for all the sets of primers we designed. Standard 100 bp ladder testify that the PCR products of GAPDH, Kv1.3, TASK-2 and KCa3.1 obtained by HWB RNA are of the expected size (117, 103, 106, and 125 bp, respectively). **(B)**: Up-regulation of K_V_1.3 mRNA in T lymphocytes of patients with multiple sclerosis. **(B1)**: Relative levels of K_V_1.3 mRNA, calculated as a ratio to GAPDH in healthy control subjects (*n* = 20) and patients with either relapsing-remitting (RRMS, *n* = 18) or secondary progressive multiple sclerosis (SPMS, *n* = 16). A statistically significant increase of K_V_1.3 mRNA expression was observed between either RRMS (1.28 ± 0.18) or SPMS (1.36 ± 0.19) and control subjects (0.76 ± 0.16). (٭٭٭: *p* < 0.001). **(B2)**: Relative TASK-2 mRNA expression in healthy controls (0.78 ± 0.12, *n* = 11), RRMS (0.79 ± 0.1, *n* = 10) and SPMS patients (0.74 ± 0.1, *n* = 9). **(B3)**: KCa3.1 expression in healthy subjects (1.12 ± 0.14, *n* = 11), RRMS (1.04 ± 0.11, *n* = 9) and SPMS (1.08 ± 0.13, *n* = 9). TASK-2 and KCa3.1 mRNA expression did not differ significantly between controls, RRMS and SPMS (ns: non-significant). All values are mean ± SE. **(C)**. Higher expression of Kv1.3 channels detected by flow cytometry in the peripheral blood T cells of patients with MS.** (** **C1)**: Kv1.3 channel expression by T lymphocytes was determined in parallel by whole-cell patch clamp (y axis) and by flow cytometry (x axis). R^2^ = 0.79. **(** **C2)**: Kv1.3 expression levels measured by flow cytometry in T lymphocytes isolated from the peripheral blood of healthy controls (squares), and patients with MS. A statistically significant increase of Kv1.3 expression was observed in T cells from MS patients compared to controls (D value = 0.22 ± 0.04 vs 0.37 ± 0.06; *p* < 0.001).

Following the functional studies that showed a specific up-regulation in Kv1.3 current responses in MS, we went on to assess the expression levels of all potassium-channel gene products reported to be expressed in human T cells, namely, Kv1.3, KCa3.1 and TASK-2 in MS patients and control subjects. We performed semi-quantitative PCR using as reference the mRNA of the housekeeping gene GAPDH. As shown in Figure 4A, PB T lymphocytes from both MS patients and controls yielded specific band products of the predicted size for all three potassium channels tested. Interestingly, T lymphocytes of MS patients had significantly higher expression levels of Kv1.3 mRNA (mean expression ratio: 1.32 ± 0.18, *n* = 34) as compared to T lymphocytes of healthy individuals (0.76 ± 0.16, *n* = 20, *p* < 0.001) (Figure 4Β). Thus, MS T cells exhibit a parallel increase of Kv1.3 mRNA expression and of the number of functional Kv1.3 channels.

Additionally, Kv1.3 mRNA expression within MS sub-groups was slightly higher in SPMS patients (1.36 ± 0.19, *n* = 16) compared to those with RRMS (1.28 ± 0.18, *n* = 18), but the difference did not reach statistical significance (Figure 4B1).

We did not detect any significant differences in the expression levels of KCa3.1 (control 1.12 ± 0.14, MS 1,06 ± 0.11) and TASK-2 (controls 0.78 ± 0.12, MS 0.77 ± 0.1) mRNA products (Figures 4B2,B3). Therefore, based on our data MS patients present a specific up-regulation in the constitutive expression of the Kv1.3 gene product.

Our data do not support previous findings reporting increased TASK-2 mRNA in MS (Bittner et al., 2010). This discrepancy could reflect differences in the experimental methods and in the set of primers used by the two groups. At this point it is worth mentioning that initially, we tried the same pair of primers for TASK-2 mRNA detection used in the aforementioned study (Bittner et al., 2010); however, in our hands these primers did not yield any detectable products at the expected size neither from mRNA isolated from human T lymphocytes nor from commercially available human brain total mRNA (TAKARA).

### Enhanced Levels of K_V_1.3 Membrane-Protein Expression in a Second MS Cohort

In a cohort of MS patients from North America, cell surface expression of K_V_1.3 was measured in freshly isolated peripheral blood mononuclear cells using a validated fluorescein-conjugated ShK-F6CA assay. ShK-F6CA blocks K_V_1.3 with low picomolar affinity and exquisite selectivity by binding to the channel tetramer (Beeton et al., 2003). The intensity of ShK-F6CA staining reflects the number of Kv1.3 tetramers on the cell surface (Beeton et al., 2003). We found a strong correlation between the mean fluorescent intensity of ShK-F6CA staining with the number of K_V_1.3 channels/cell determined by whole-cell patch clamp (Figure 4C1). Peripheral blood mononuclear cells from North American MS patients expressed higher ShK-F6CA staining than cells from healthy individuals, which is indicative of more cell surface K_V_1.3 tetramers (Figure 4C2, *p* < 0.001).

## Discussion

In this study we report that T lymphocytes of MS patients specifically express higher levels of the voltage-gated potassium channel Kv1.3 than their control group counterparts. This Kv1.3 up-regulation in MS is evident at the cell surface protein level, the density of functional channels and at the amount of mRNA.

These results are at odds with an earlier report that presented TASK-2 specific up-regulation in T cells of MS patients (Bittner et al., 2010). Our experiments diminish the possibility of a TASK-2 contribution in our potassium current recordings and fail to show the presence of any detectable functional TASK-2 channels in freshly isolated unsorted resting human T lymphocytes under our experimental conditions that differ in the composition of the recording solutions, the voltage protocols (holding potential, inter-pulse duration) and the use of sorted (CD4^+^ and CD8^+^ T cells) vs unsorted T cells. This finding is consistent with other reports that either failed to record TASK-2 currents or report that the only potassium channels in human and rat T cells are Kv1.3 and KCa3.1 (Hajdú et al., 2010; Chiang et al., 2017). Additionally, we show that although TASK-2 mRNA is constitutively expressed in PB T lymphocytes, it is only the Kv1.3 mRNA that is up-regulated in MS. These discrepancies however, may merely reflect differences in the experimental conditions (primers, cycles or enzymes) or the T cell subpopulation tested (MACS-sorted CD8^+^ PB T cell vs unsorted PB T cells).

Earlier studies have demonstrated the specific presence of myelin auto-reactive T_EM_ cells in the PB of MS patients (Wulff et al., 2003), that when activated acquire a Kv1.3^high^ phenotype. Therefore, it is only just for someone to wonder if in our experiments, recordings from a few Kv1.3^high^ T lymphocytes could have skewed our average data to show increased Kv1.3 channel density in MS. However, according to our data more than 85% of MS T lymphocytes had augmented Kv1.3 current responses; moreover the majority of clamped cells had a small size (1,6 pF), consistent with resting rather than activated T lymphocytes. Therefore, our data support a global increase of Kv1.3 channel density in resting T lymphocytes of MS patients and do not seem to concern a minor subpopulation of these cells because otherwise we would not be able to detect it. This increase goes along with a concomitant amplification in the constitutive levels of Kv1.3 mRNA, suggesting that the up-regulation of Kv1.3 channels in MS is the product of an enhanced gene transcription rather than of a translational or post-translational modification. This could be the result of either genetic or epigenetic factors and warrants further investigation.

The finding of a specific Kv1.3 up-regulation in the PB T lymphocytes of patients with MS, complements a long line of evidence that strongly supports Kv1.3 channel involvement in MS pathophysiology, such as: the presence of Kv1.3^high^ T_EM_ cells in the inflammatory lesions of MS brains (Rus et al., 2005); the association of a Kv1.3 gain-of-function gene polymorphism with MS severity (Lioudyno et al., 2021); the Kv1.3 up-regulation in brains of animals with experimental allergic encephalomyelitis (Bozic et al., 2018); the finding that Kv1.3 pharmacological blockade or Kv1.3 gene-silencing in animal models of MS 1) renders mice resistant to experimental allergic encephalomyelitis (Beeton et al., 2001), 2) decreases demyelination (Murray et al., 2015), and 3) drives T cells toward an immune-regulatory phenotype (Gocke et al., 2012).

**FIGURE 5:**
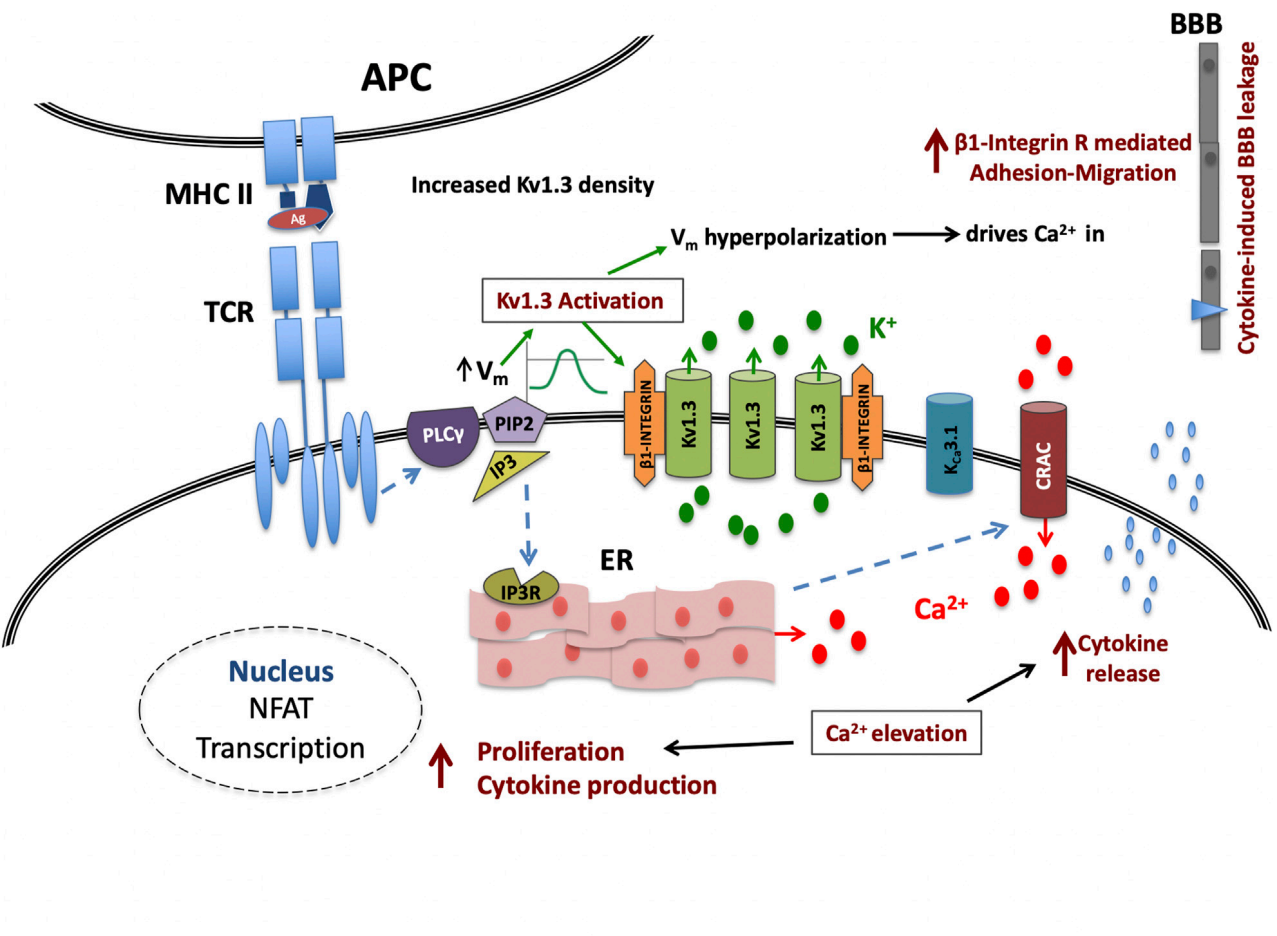
Kv1.3 channel up-regulation enhances signaling in MS T cells. Upon TCR antigenic (Ag) stimulation, the IP3-induced calcium release from the intracellular stores (ER) causes on one hand membrane depolarization (Vm), followed by a Kv1.3 channel-mediated hyperpolarization and on the other, the formation of conductive Calcium Release Activated Calcium (CRAC) channels; in MS-T cells this hyperpolarization will be of increased magnitude due to higher Kv1.3 channel density. This in turn will translate into a stronger than physiological driving force for calcium ions (Ca^2+^) through CRAC channels and therefore a cytosolic calcium signal of amplified intensity and duration that in turn will potentiate the effectiveness of the immune stimulus, and may even allow sub-threshold stimuli to reach activation threshold, priming T cell activation that otherwise would not. Additionally all calcium-dependent processes that follow the successful activation of MS T cells, such as cytokine production, cytokine secretion and proliferation, will be heightened. Moreover, the immune stimulus-induced Kv1.3 activity in these cells, will increase β1-integrin activation, facilitating their adhesion and cytokine-induced migration into the CNS.

The pathophysiological relevance of the increase in the density of active Kv1.3 channels in T lymphocytes of MS patients can only be evaluated if we take into account the role of these channels in T lymphocyte processes that determine the fate and function of these cells. As aforementioned, Kv1.3 are the main potassium channels of T lymphocytes at rest and during the initial steps of the activation process and their functional relevance is underscored by their localization in the immune synapse (Panyi et al., 2004) that fulfills the spatiotemporal requirements for their participation in the activation-signaling network. Activation of Kv1.3 channels generates graded negative membrane potentials proportional to the potassium ions flowing out of the cell. Membrane hyperpolarizations are of crucial biological importance for the activation of T lymphocytes because they provide the necessary negative electromotive force that enables calcium ions to flow into the T lymphocytes through CRAC channels, thus sustaining the cytosolic calcium elevations in order for the activation process to proceed. From the above it becomes evident that the activity of Kv1.3 channels is directly linked to the amount of calcium ions that enter the cell. On the other hand, the activity of Kv1.3 is dictated by the density of functional channels on the plasma membrane and by their opening probability, which is governed by the degree of membrane depolarization. Consequently, similar antigenic stimulations in resting T lymphocytes of the same subset will produce calcium signals of different magnitude and duration when membrane densities of functional Kv1.3 channels vary (Figure 5).

Based on the above, the Kv1.3 up-regulated phenotype of T lymphocytes could account for and is consistent with the previously reported enhanced calcium responses (Martino et al., 1998) and T cell hyper-responsiveness in MS. The predicted increase of calcium influx through CRAC channels in MS T cells, is expected to enhance the production of proinflammatory cytokines (e.g. interferon-γ, interleukin-17 and Tumor Necrosis Factor α), known to be associated with MS pathogenesis (Wagner et al., 2020), via calcineurin-NFAT (Nuclear factor of Activated T cells) activation (Gwack et al., 2007); it is also expected to render these cells less dependent on co-stimulatory signals (Markovic-Plese et al., 2001) and more resistant to co-inhibitory ones (Fife and Bluestone, 2008) and therefore to be one of the underlying factors that drive T cell function in MS away from a context-specific responsiveness and immune homeostasis.

Stronger hyper-polarizations following T lymphocyte stimulation in MS, would act as signal enhancers and thus buoy weak stimuli (low affinity antigens, low antigen titters or antigen peptides with low homology to the cognate ones) to reach activation threshold. The propensity for immune activation by the Kv1.3 up-regulation in MS may at least partly explain the presence of activated T cells, especially the myelin-specific ones (Zhang et al., 1994) that characterise this disorder. Moreover, activation of Kv1.3 channels induces β1-integrin receptor activation and promotes T cell adhesion and migration (Levite et al., 2000) and Kv1.3 channels have been shown to modulate T cell motility within inflammatory tissues (Matheu et al., 2008). Thus increased Kv1.3 expression will favor trafficking of MS T cells to inflammatory sites within the CNS, where they may contribute to myelin destruction.

The increased Kv1.3 channel density in MS could negatively interfere with the immune mechanisms responsible for deleting potentially pathogenic T cells from the periphery in order to sustain immune homeostasis and avert autoimmunity. Namely, the CD95/Fas apoptotic pathway has Kv1.3 down-regulation as one of the first steps (Szabo et al., 1996); consequently Kv1.3 up-regulation may confer a degree of resistance to Fas-induced apoptosis in MS T-lymphocytes. Aberrations in this pathway have been linked to autoimmunity and more specifically to MS (Volpe et al., 2016).

From the above it is valid to argue that T lymphocyte Kv1.3 up-regulation in MS may be an important factor in disease pathogenesis. Strikingly, we found significantly higher Kv1.3 channel expression in SPMS compared to RRMS; the underlying cause of this finding cannot be explained by our data and warrants further investigation. It could represent a disease stage-specific enhancement in the functional expression of Kv1.3 channels due to different levels of systemic immune activation; interestingly it has been reported that SPMS T lymphocytes show significantly reduced Fas-mediated apoptosis compared to RRMS (Comi et al., 2000), a finding that could be accounted for by the enhanced Kv1.3 channel expression in SPMS. Alternatively, based on a recent genetic study that as aforementioned, associates a gain-of-function Kv1.3 gene polymorphism to a more aggressive disease course (Lioudyno et al., 2021), one may propose that the higher Kv1.3 expression in the SPMS group could merely reflect a history of more active disease tending to progress to the SPMS phenotype with a higher frequency.

In summary, our study is in line with a body of evidence showing that Kv1.3 channels may be involved in MS and supports the pharmacological inhibition of their activity as a potential treatment for the disease, especially for SPMS, where treatment options are limited. Moreover, the specificity of Kv1.3 up-regulation for MS compared to other neurological disorders with or without inflammation, strongly supports the use of Kv1.3 expression as a peripheral biomarker for the disease.

## Data Availability

The original contributions presented in the study are included in the article/Supplementary Material, further inquiries can be directed to the corresponding author.
